# Supplementary material for: Association between oral microbiome and five types of respiratory infections: a two-sample Mendelian randomization study in east Asian population
Source: Front Microbiol. 2024 Apr 10;15:1392473. doi: 10.3389/fmicb.2024.1392473 (PMC11039966; doi:10.3389/fmicb.2024.1392473)
Supplement: Supplementary file 1 [file Data_Sheet_1.zip › Supplementary Materials/MR plots of saliva/Pneumonia/s__Neisseria_sp000186165_mgs_3225/forest.pdf]

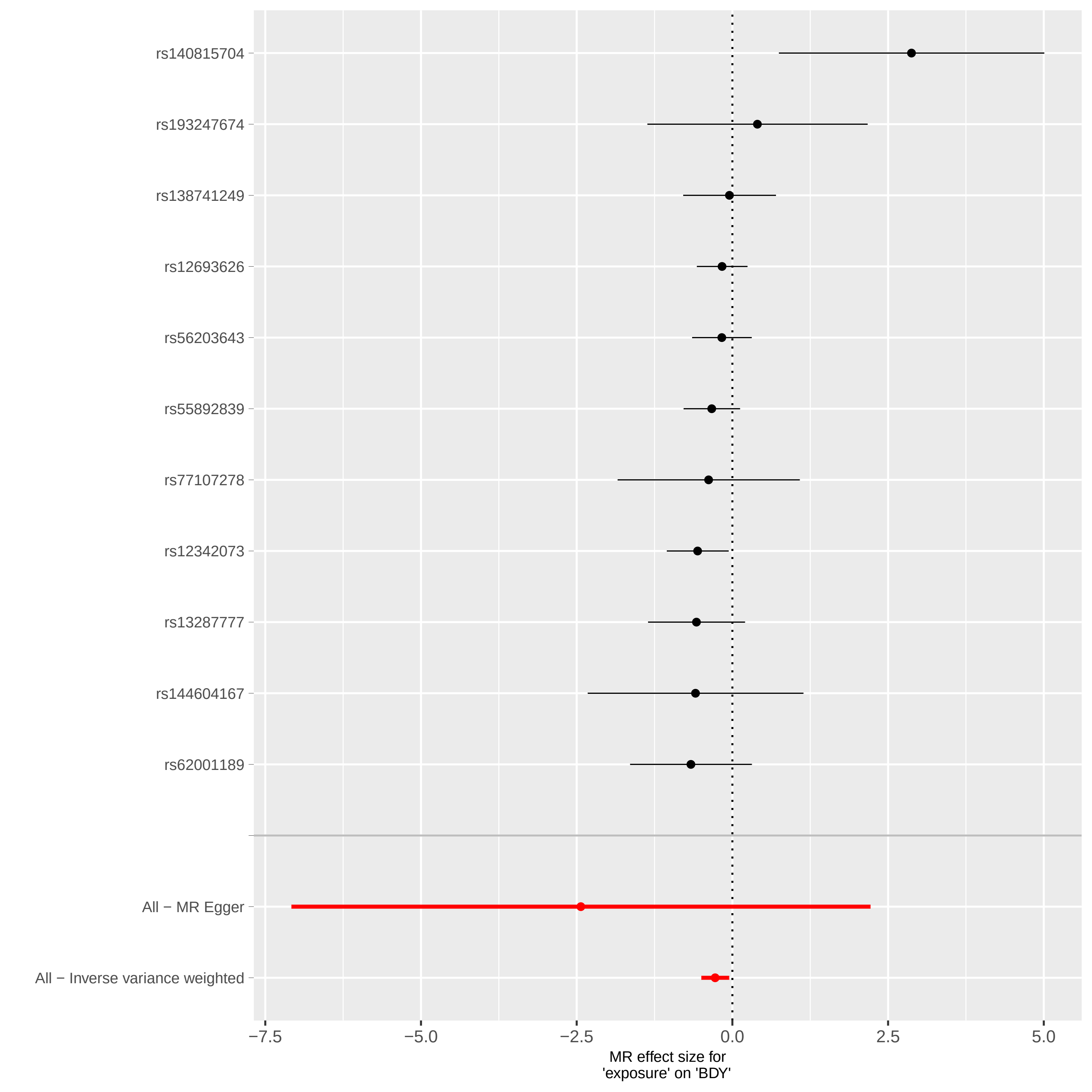

rs140815704
rs193247674
rs138741249
rs12693626
rs56203643
rs55892839
rs77107278
rs12342073
rs13287777
rs144604167
rs62001189
All − MR Egger
All − Inverse variance weighted
−7.5
−5.0
−2.5
0.0
2.5
5.0
MR effect size for
'exposure' on 'BDY'
